# Implementing a National Electronic Referral Program: Qualitative Study

**DOI:** 10.2196/10488

**Published:** 2018-07-18

**Authors:** Marcella McGovern, Maria Quinlan, Gerardine Doyle, Gemma Moore, Susi Geiger

**Affiliations:** ^1^ Applied Research for Connected Health University College Dublin Dublin Ireland; ^2^ UCD College of Business University College Dublin Dublin Ireland

**Keywords:** electronic referrals, scale-up, eHealth, implementation, policy

## Abstract

**Background:**

Electronic referrals or e-referrals can be defined as the electronic transmission of patient data and clinical requests between health service providers. National electronic referral systems have proved challenging to implement due to problems of fit between the technical systems proposed and the existing sociotechnical systems. In seeming contradiction to a sociotechnical approach, the Irish Health Service Executive initiated an incremental implementation of a National Electronic Referral Programme (NERP), with step 1 including only the technical capability for general practitioners to submit electronic referral requests to hospital outpatient departments. The technology component of the program was specified, but any changes required to embed that technology in the existing sociotechnical system were not specified.

**Objective:**

This study aimed to theoretically frame the lessons learned from the NERP step 1 on the design and implementation of a national health information technology program.

**Methods:**

A case study design was employed, using qualitative interviews with key stakeholders of the NERP step 1 (N=41). A theory-driven thematic analysis of the interview data was conducted, using Barker et al’s *Framework for Going to Full Scale*.

**Results:**

The NERP step 1 was broadly welcomed by key stakeholders as the first step in the implementation of electronic referrals—delivering improvements in the speed, completeness of demographic information, and legibility and traceability of referral requests. National leadership and digitalized health records in general practice were critical enabling factors. Inhibiting factors included policy uncertainty about the future organizational structures within which electronic referrals would be implemented; the need to establish a central referral office consistent with these organizational structures; outstanding interoperability issues between the electronic referral solution and hospital patient administration systems; and an anticipated need to develop specialist referral templates for some specialties. A lack of specification of the sociotechnical elements of the NERP step 1 inhibited the necessary testing and refinement of the change package used to implement the program.

**Conclusions:**

The key strengths of the NERP step 1 are patient safety benefits. The NERP was progressed beyond the pilot stage despite limited resources and outstanding interoperability issues. In addition, a new electronic health unit in Ireland (*eHealth Ireland*) gained credibility in delivering national health information technology programs. Limitations of the program are its poor integration in the wider policy and quality improvement agenda of the Health Service Executive. The lack of specification of the sociotechnical elements of the program created challenges in communicating the program scope to key stakeholders and restricted the ability of program managers and implementers to test and refine the change package. This study concludes that while the sociotechnical elements of a national health information technology program do not need to be specified in tandem with technical elements, they do need to be specified early in the implementation process so that the change package used to implement the program can be tested and refined.

## Introduction

### Electronic Referrals

Electronic referrals or e-referrals can be defined as the electronic transmission of patient data and clinical requests between health service providers [1]. Shifting from paper-based referrals (ie, postal letter or fax) to electronic referrals offers the opportunity to transform the interface between primary and specialty care [2]. Historically, the default clinical request from a general practitioner (GP) referring to a specialist was to request a face-to-face consultation with a given service user [3]. Electronic referral technology, however, can support a two-way channel of communication between referrer and referee, creating the opportunity for more flexible and consultative forms of data exchange and clinical requests [4]. In addition, electronic referrals provide health systems with the capability to optimize system capacity, whereby GPs can be supported by specialists to care for service users in the community until they genuinely require a specialist appointment [5].

### Development of Electronic Referrals in the Irish Health Service Executive

The initiation of an Irish electronic referral pilot program in January 2011 was not solely motivated by the potential for electronic referrals to transform the interface between primary and specialty care. A crisis emerged in March 2010, when the media reported that one of Ireland’s largest hospitals had 30,000 unopened or unprocessed GP outpatient referrals. The Irish Health Service Executive (HSE) commissioned an investigation [6] and the Health Information and Quality Authority (HIQA) partnered with the Irish College of General Practitioners (ICGP) to conduct a review of the referral management between GPs and hospital outpatient departments (OPDs; GP-OPD). This HIQA-ICGP partnership created a standardized general referral template, specifying the essential information that needs to be contained in a referral from a GP to a hospital OPD. In addition, the HIQA-ICGP partnership recommended that their standardized template could form the informational basis for an electronic referral solution between GPs and OPDs [7].

Meanwhile, an advisory group had been established in the South of the country, comprising clinical, management, information technology (IT), and patient representatives, to reconfigure hospital services in that region. The group partnered with *Healthlink*—an Irish structured health care messaging platform—to develop and pilot an electronic referral pathway between GPs and OPDs for 7 hospitals in their region. The pilot project revealed several challenges for implementing an end-to-end electronic referral solution, capable of offering a two-way interface between GPs and hospital OPDs. Foremost of these challenges were the outstanding interoperability issues between the *Healthlink* platform and hospital patient administrative systems (PAS); second, the human resourcing of hospital central referral offices (CROs) to process electronic referrals.

Despite these obstacles to the implementation of an end-to-end electronic referral solution, the pilot project successfully established the technical capability, through the *Healthlink* platform, for GPs to electronically submit their referrals to hospital OPDs. This first step in the electronic referral process has been described as the electronic referral request [8]. Furthermore, the pilot project found that the use of electronic referral requests resulted in the following improvements: (1) improved legibility because all information is typed in a standardized template, (2) improved completeness of data because of the mandatory fields in the standardized template, (3) assurance for GPs and patients that their referral had been received because an automated email is returned to the referring GP once it is digitally opened in the hospital, and (4) improved traceability and visibility for hospitals in the referral management because *Healthlink* creates a digital record of when and how many electronic referrals have been received by each hospital, and when they were triaged [9].

These simple and yet important patient safety benefits informed a decision by a newly established unit in the HSE, called *eHealth Ireland*, to establish a National Electronic Referral Programme (NERP) with step 1 involving the scale-up of the technical capability for GPs to submit electronic referral requests to hospital OPDs.

### Impact of Scale on a Sociotechnical Approach

Reviews from some of the earliest deployments of national electronic referral systems, including Norway [10], the Netherlands, and Denmark [11], recommend a sociotechnical approach to implementing electronic referrals. A sociotechnical approach considers how the technical features of a health information system interact with the social features of a health care work environment [12]. *eHealth Ireland* ’s decision to initiate the NERP on an incremental basis, that is, step 1 with only the technical capability for electronic referrals, appears to be at odds with this sociotechnical approach. The only target specified was to establish, within 12 months, the technical capability for GPs to submit electronic referral requests to at least one OPD specialty in all hospitals. Regarding the adoption of that technical capability, no targets were set for which OPD specialties would be included in the program, what the target volume of electronic referrals versus paper referrals would be, or what proportion of GPs would be engaged. That is, the technology component of the program was specified, but the extent at which that technology would interact with the sociotechnical system was not specified.

In the UK, Eason applied a sociotechnical lens to the implementation of the National Health Service’s National Programme for IT and highlighted that in national health IT programs, the desired technical and social systems are not designed and implemented simultaneously [13], as suggested by many interpretations of a sociotechnical theory approach [14].

**Figure 1:**
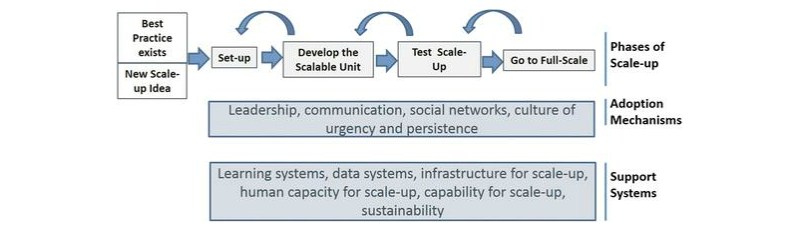
Theoretical framework—Barker et al’s [16] *Framework for Going to Full Scale* (reprinted with permission from P Barker).

Instead, standard technical systems are predefined at the national level, and flexibility needs to be provided for local implementation sites to adopt technical systems in ways that meet local needs and enable them to engage in sociotechnical systems design at a level where the local user community can play a full part [13]. This suggestion that the sociotechnical approach needs to be modified for national health IT programs is reflected in the design of the NERP step 1 [15], where the technical system was predefined nationally, but it was left to local implementation sites to undertake the sociotechnical systems design work. However, it remains unclear from Eason’s critique of sociotechnical systems theory if or when the sociotechnical elements of a national health IT program should be defined at the national level [13].

### Study Goal

To contribute to this discussion on the design and implementation of national health IT programs, this paper presents the findings from qualitative, in-depth interviews conducted with key stakeholders in the implementation of the NERP step 1. The following two research questions seek to explore the arguments for and against progressing with the scale-up of only the technical elements of a national health IT program, using Ireland’s NERP step 1 as an empirical case study: (1) What were the strengths and limitations of the scale-up of the NERP step 1, as a technical-only intervention? and (2) Do the sociotechnical elements of a national health IT program need to be specified at the national level?

This study aims to theoretically frame the lessons learned from Ireland’s NERP step 1 for policy makers and implementers seeking guidance on how to design and implement national health IT programs.

### Theoretical Framework

We adopted the Institute for Healthcare Improvement’s *Framework for Going to Full Scale* [16] as the theoretical framework to guide our empirical inquiry and analysis. This framework proposes that to take a health care quality improvement to full scale, it is first necessary to account for the factors required to promote the adoption of changes and support scale-up, and second, to design at the outset a phased plan to reach full-scale implementation.

The *phases of scale-up* proposed by this framework for health care quality improvement include: (1) Set-Up; (2) Develop the Scalable Unit; (3) Test Scale-Up; and (4) Go to Full Scale. Each of these phases is either enabled or inhibited by the availability of certain *adoption mechanisms* and *support systems* (Figure 1). *Adoption mechanisms* to account for include better ideas, leadership, communication, policy, and a culture of urgency and persistence. *Support systems* include human capability for scale-up, infrastructure for scale-up, data collection and reporting systems, a learning system, and the need to design for sustainability.

An important reason for selecting this theoretical framework is that it can accommodate the NERP’s incremental design, whereby this study only examines step 1 in the implementation of electronic referrals and not the complete implementation of electronic referrals. The *phases of scale-up* in this framework are informed by Plan-Do-Study-Act (PDSA) cycles of quality improvement. It is not assumed that what is being implemented is the complete program. A PDSA cycle requires only that for any given program or program component, a theory of change can be specified and then tested across a range of contexts before being implemented at full scale. The framework contains a feedback loop so that the first three *phases of scale-up* can be revisited and adapted if new learnings at a later phase reveal that an adaptation would optimize the scale-up. Figure 1 presents this feedback loop by the counter-clockwise arrows above the four *phases of scale-up*.

## Methods

### Methodological Approach

This study explored the implementation of the NERP step 1 using qualitative, in-depth interviews with key program stakeholders. This approach captures individual participants’ experiences, narratives, ideas, and discourses [17] and informs an analysis of the scale-up strategy employed.

### Recruitment

#### Ethics

Ethical approval was granted by the Office of Research Ethics in University College Dublin (UCD). No vulnerable populations participated in this study, and no patient data were collected. All participants were interviewed in a professional capacity as stakeholders in the scale-up of electronic referrals in Ireland. Participant anonymity and confidential data management were the dominant ethical considerations for this study and were maintained in line with UCD Research Ethics Guidelines.

#### Participants

This study investigated the lessons to be learned from the NERP step 1 on scaling-up only the technical elements of a national health IT program. Access to 1 of 7 pilot sites and 5 of 42 sites targeted by the NERP step 1 was facilitated by *eHealth Ireland*. Although not randomly selected, the 5 NERP step 1 sites included public and voluntary hospitals, as well as regional and urban hospitals, providing a broad representation of implementation sites. The key inclusion criterion for recruiting participants was as follows: Has this stakeholder been involved in the design or implementation of the NERP step 1? If not, it was considered unsuitable for stakeholders to serve as key informants on the strengths or limitations of a technical-only scale-up or in offering an empirical assessment of whether the sociotechnical elements of a national health IT program should be specified at the national level. Based on this criterion, we did not include service users and hospital specialists in our study design because they were not directly involved in designing or implementing the program at this early step 1 stage of the NERP. However, studies of later stages of the implementation should include these crucial stakeholders, where, for example, service users might have access to a Web-based appointment portal, or hospital specialists might be engaged to design specialist referral templates and therefore, will be in a position to speak about their experiences of designing and implementing the program.

The following participants were recruited from the pilot site: pilot management (n=3); hospital administration or management (n=3); general practice (n=3); and information communication technology (ICT; n=3). In addition, the pilot general practice and ICT stakeholders were involved in the NERP step 1 and therefore appear in Table 1 as both pilot and NERP national-level stakeholders. Moreover, the NERP national-level stakeholders included NERP management (n=2) and other HSE stakeholders (n=3) who were involved in the design and implementation of the NERP step 1. At the implementation site level, we recruited 4 additional general practice stakeholders, including 3 GPs and 1 general practice secretary, bringing the number of GPs who participated in the study to 6 out of approximately 3000 GPs [18] operating in the Irish health system. Furthermore, participants recruited from within the NERP step 1 hospital sites included the following: hospital administration or management (n=17) and hospital ICT (n=3). As a qualitative study, participants (N=41) were recruited as key stakeholders and informants on the design and implementation of the NERP step 1; they did not constitute a representative sample of their peers in the Irish health system.

Overall, 28 interviews were scheduled. Of 41 participants, 19 participated in face-to-face group interviews and the remaining 22 were interviewed individually. The group interviews involved a range of 2-5 participants and were predominantly undertaken with the hospital administration or management stakeholder group. Of 22 interviews conducted with individual participants, 5 were conducted via telephone and 17 on a face-to-face basis. All participants consented to have their interview recorded at the outset, using a digital voice recorder.

### Data Analysis

We conducted a thematic analysis, using Braun and Clarke’s [19] 6-phase procedure for thematic analysis and Barker et al’s [16] framework to organize the data from a scale-up perspective. Thematic analysis is a method for identifying, analyzing, and reporting patterns (themes) within data [19]. The audio recordings of all 28 interviews were transcribed by the interviewing author (GM). Thematic analysis does not require a full verbatim transcription, including nonverbal cues (eg, silences, body language, and external noises) or emotional aspects (eg, laughs, coughs, and sighs) [20]. Such data would have contributed little toward answering this study’s research questions but would have taken a substantial amount of time and resources. All transcripts were cross-checked by the lead author (MMG) who audited transcription by listening through all audio recordings while reading the transcripts.

**Table 1:** Participant involvement in the National Electronic Referral Programme (NERP; N=41).

Key stakeholder types		Involvement, n (%)		
		Pilot (n=12)	NERP (n=35)	Both (n=6)
**Pilot**				
	Pilot management	3 (25)	—	—
	Hospital administration or management	3 (25)	—	—
**NERP national level**				
	NERP management	—	2 (6)	—
	Health Service Executive	—	3 (8)	—
	Information communication technology	3 (25)	3 (8)	3 (50)
	General practice	3 (25)	3 (8)	3 (50)
**NERP implementation sites**				
	Hospital administration or management	—	17 (49)	—
	Information communication technology	—	3 (8)	—
	General practice	—	4 (11)	—

Furthermore, any points of divergence in the interpretation of how the spoken word should be written in the transcripts were documented and later discussed by MMG and GM to obtain an agreement on transcription.

In the first analytical step, we conducted an inductive thematic analysis [19]. Overall, 149 initial codes were generated and collated into 7 initial themes as follows: (1) Stakeholder Consultation (12 codes); (2) Scope and Pace of Change (15 codes); (3) Technological Design (31 codes); (4) Organizational Change (25 codes); (5) Engagement (18 codes); (6) Quality Improvement (37 codes); and (7) Irish Context (11 codes). The two research questions aimed to explore the arguments for and against progressing with the scale-up of only the technical elements of a national health IT program. Among the diverse stakeholders interviewed, contradictory perspectives were articulated on whether or not it was the correct decision to progress to the large-scale implementation with a technical-only solution.

In the second analytical step, Barker et al’s *Framework for Going to Full Scale* was identified as a framework suitable for guiding a more theoretically driven thematic analysis [19] of the data, which could then be overlaid with the inductive coding. Coding and analyzing the data within this framework’s *phases of scale-up*—each of which was either enabled or inhibited by specific *adoption mechanisms* and *support systems*—produced a coherent and accurate representation of the diverse perspectives expressed in the data. An initial, comprehensive report was produced by MMG, using the data coded under each theme to present a theory-driven response to the two research questions. Through an iterative process of review and revisions involving first, MQ and GM, and then GD and SG, the authors reached consensus on the final structure of the findings and the selection of representative quotes. This collaborative process facilitated an investigation of any contradictory evidence or possible alternative interpretations of the data to ensure the minimization of individual bias in the results presented.

## Results

### Data Presentation

**Figure 2:**
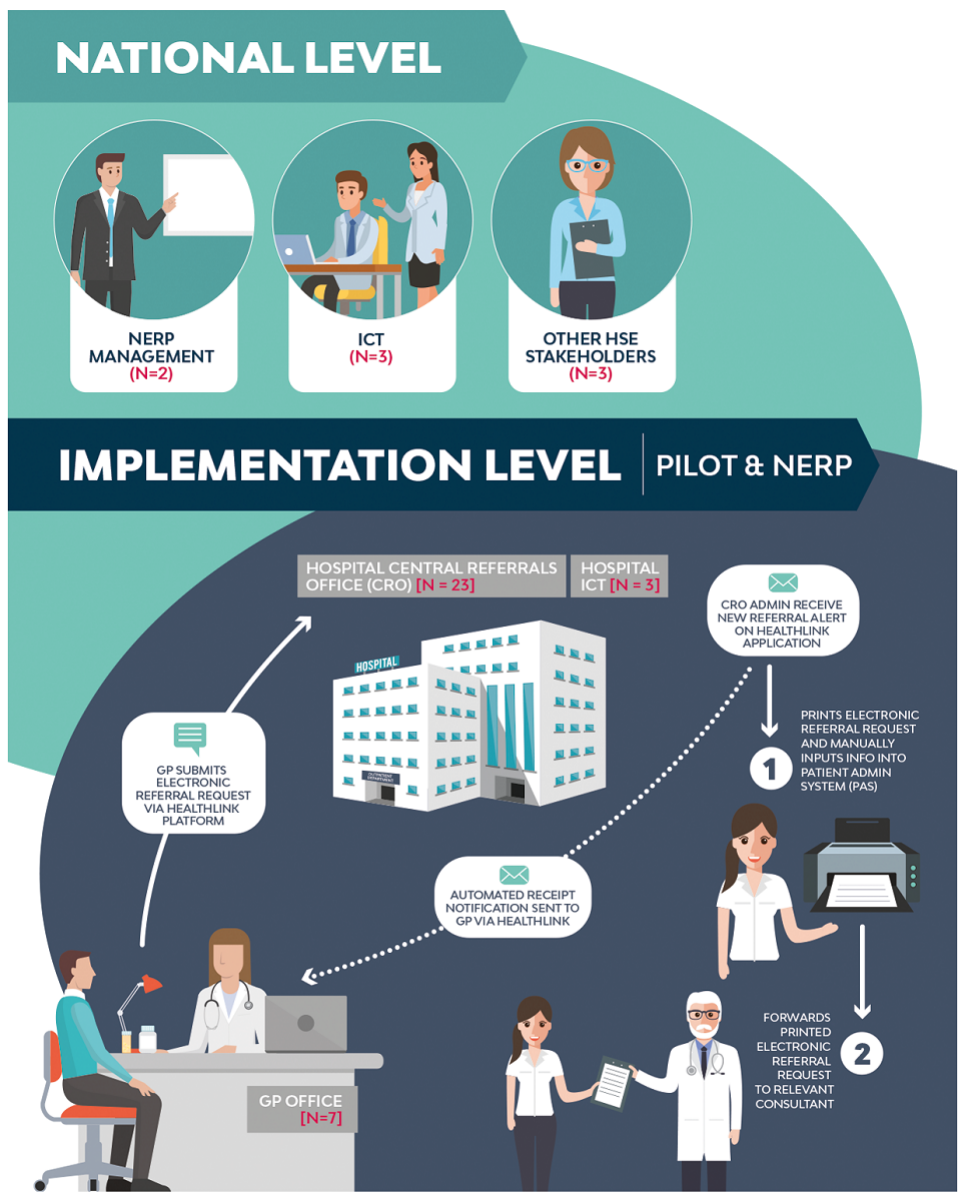
The stakeholder map. GP: general practitioner, ICT: information communication technology, NERP: National Electronic Referral Programme.

This section presents data collected via qualitative in-depth interviews with key stakeholders of the NERP step 1 using the theoretical structure provided by Barker et al’s *Framework for Going to Full Scale*. Figure 2 illustrates a stakeholder map of the number and type of stakeholders, who informed these results and where they come in the process flow of the NERP step 1.

**Figure 3:**
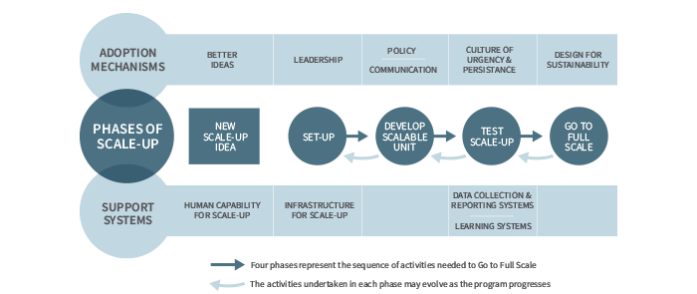
Application of the *Framework for Going to Full Scale* to the NERP step 1.

Figure 3 illustrates the application of *Framework for Going to Full Scale* to this study’s data, by indicating the *phases of scale-up* that each adoption mechanism and support system emerged in the analysis, as an enabler or inhibitor of scale-up.

Finally, to provide some context for these results, Multimedia Appendix 1 charts the number of electronic referrals submitted to the pilot hospital OPD and the 5 NERP hospital OPDs who participated in this study in 2015 and 2016, when NERP step 1 was implemented.

### New Scale-Up Idea

The *phases of scale-up* are triggered by the discovery of a *new scale-up idea* or a new best practice, which is perceived by the stakeholders as a “better idea” (*adoption mechanism*). A national-level participant commented that the NERP step 1 was:

...an easy sell, [its] patient safety...a solution has been developed so it’s a matter of taking the solution and rolling it out to different acute hospitals.

Participant 28

Similar to the pilot experience, the NERP step 1 participants cited speed, complete demographic information, legibility, and traceability as the 4 key patient safety improvements delivered in the NERP step 1. Participants commented that *“speed of referral would be the biggest thing...The GP knows it’s got here”* (Participant 10) because an automated notification is returned to the GP once the electronic referral has been opened within the hospital.

It’s instant, it sells itself. You send in the referral, the hospital has it, there’s no post, you’re not waiting a day for it to be delivered.

Participant 29

It’s good they have a minimum data set...Like we’ll never be missing a date of birth or [receive]...only one line of an address...They will always give you a phone number on it.

Participant 40

Another hospital administrator pointed out that full contact details, including mobile phone numbers, are very important so that they can *“text remind people...to help reduce the DNA* (Do Not Attend) *rate”* (Participant 5). Finally, several participants commented on the legibility benefits of electronic referrals in that they save time trying to decipher difficult hand-writing or calling GP surgeries to confirm details or to seek missing information.

It’s legible you know. Many times you have to ring them up [GP surgeries].

Participant 40

However, with electronic referrals, they *“can find a patient much easier on the system now”* (Participant 24).

One important sociotechnical element built into the pilot program had been the requirement for hospitals to return a triage outcome message, via *Healthlink*, to referring GPs. Manpower planning issues identified in hospital CROs during the pilot stage resulted in the GP triage outcome message not being specified as an element of the NERP step 1. One of the pilot GPs commented that:

...it remains to be seen...how negative that will be...It reduces the communication back to the GP, and it doesn’t tell the GP how the patient has been triaged.

Participant 16

A national-level participant commented that:

...we thought the GPs would be up in arms and they would go crazy about it but actually when we did go back to the ICGP...they said, “we’d be disappointed but at the same time...we would prefer that they [hospitals] went with it without responses than not go at all.”

Participant 12

As such, GPs supported the NERP step 1 in proceeding with the scale-up of the technical capability for all hospitals to receive electronic referral requests, with the knowledge that hospitals did not have the “human capability for scale-up” (*support system*) to support an electronic processing of referrals (ie, eTriage and eAppointments). This acceptance among key stakeholders that the NERP step 1 is only about paving the way for the complete implementation of electronic referrals is also captured in a comment by a national-level participant who described the NERP step 1 as getting: “...the footprint of eReferrals out to all hospitals around the country” (Participant 18).

### Set-Up

Set-Up is the first *phase of scale-up*, where the ambition for full scale is defined. Limiting the scope of the NERP step 1 to the technical capability was explained by national-level participants as a pragmatic decision. A national-level stakeholder explained that they did not *“have the bandwidth within* [their] *resources to go to each site”* to support a sociotechnical implementation.

When we are asked to rollout eReferrals within 12 months, what we can do is we can put the capability in place for each of the sites.

Participant 18

In terms of the “ask” to rollout electronic referrals in 12 months, the “leadership” provided by the HSE’s new *eHealth Ireland* unit was widely cited as a critical *adoption mechanism* for the set-up of the NERP. One pilot participant suggested that the *“timing was impeccable”* (Participant 28) for the appointment of a chief information officer (CIO) to lead the *eHealth Ireland* unit.

If he hadn’t arrived I would say that at this stage, we’d have rolled it out in the South or Southwest Hospital Group [pilot] and possibly no further.

Participant 28

A national-level participant commented that:

...this is the first...of any of the projects we’ve done, where there’s been a national focus. Where from the top, it’s been said, “everyone has to accept electronic referrals by X date.”

Participant 12

In addition, a GP who was involved in the pilot commented that the new CIO was *“providing a vision for where the service needs to go”* but...

...there’s a huge amount of work that needs to be done and huge investment that needs to take place, and I suppose that remains to be seen, whether that will be available.

Participant 16

This last comment suggested that leadership requires not only vision but also the ability to secure funding. A comment by a national-level stakeholder supported this suggestion in referring to the *“credibility piece,”* whereby:

...if we can deliver a project of this type in a timeline that’s considered sensible...then it provides more confidence that the Office of the CIO and the Healthlink team can actually deliver significant change in a reasonable amount of time.

Participant 18

This comment provided context for the ambitious 12-month time-frame to scale-up the technical capability for electronic referrals, particularly because further resources need to be secured to proceed beyond step 1 of the NERP.

Second, defining the ambition of the NERP step 1 as putting the technical “capability” in place for all hospitals to receive electronic referral requests from GPs indicated confidence that GPs will submit their referrals electronically, should this facility be available to them. The level of digitalization of general practice health records represents an important “infrastructure for scale-up” *support system*. The *Healthlink* solution is fully integrated into general practice software packages, and GP participants emphasized the importance of this integration in their interviews.

[GPs] have all the information in the system and being able to extract it and package it up and send it off electronically is kind of a side effect of the investment that they’ve [GPs] made over the years.

GP 1

No formal incentives are offered to GPs to use electronic referral requests and, therefore, their adoption of the solution relies upon their technical capability to submit electronic referrals and the perception that this solution is a “better idea.”

### Develop the Scalable Unit

The second *phase of scale-up* is developing the scalable unit, which is the smallest unit of the system targeted for the full-scale implementation. This analysis proposes that the scalable unit of the NERP step 1 should specify the proportion of (1) hospitals targeted, (2) OPD specialties targeted, (3) electronic versus paper referral requests targeted, and (4) GPs targeted to use electronic referral requests. In practice, the scalable unit specified by *eHealth Ireland* only included the first of these elements, with a full-scale target of all public hospitals.

Implementing this first element was the focus of the NERP step 1 throughout this study period (October 2015-May 2016) and was achieved in May 2016, after 17 months of implementation, when (1) all public hospitals had (2) at least one specialty accepting (3) outpatient electronic referrals from (4) referring GPs. With all hospitals targeted, a minimal specification of at least one OPD specialty in each hospital can be assumed. However, no specification was provided for the target proportion of electronic versus paper referral requests or the proportion of GPs targeted to use electronic referral requests. The remainder of the description of the NERP step 1’s progression through the *phases of scale-up* will, therefore, be dealing with what remains to be scaled rather than what has been scaled.

Interview data collected suggested that the incomplete development of the scalable unit reflects uncertainty in national “Policy” (*adoption mechanism*). The Irish health system is undergoing a process of de-centralization, from a highly centralized HSE to the creation of hospital groups and community healthcare organizations [21,22]. These structures are yet to be finalized [23], creating a challenge for the NERP step 1 because it is envisioned that CROs will ultimately be created at the hospital group level, rather than within individual hospitals [24]. Arguably, it would be a duplication of effort to implement the sociotechnical process changes associated with electronic referrals at the hospital level only for those processes to be changed again once there is certainty about the hospital groups. A national-level stakeholder highlighted that it is not a decision for *eHealth Ireland* whether outpatient electronic referrals will be managed in each hospital or at a hospital group level.

[It is] not something that IT can make a call on...we’ll certainly drive it once we’re clear this is a direction that is best for the patient and for the service.

Participant 18

This quote illustrated a governance challenge faced by national health IT programs like NERP, in that the authority to make key decisions about the design of such programs might lie outside of the program team.

In addition, shortcomings emerged at this phase in the “communication” *adoption mechanism*. Barker et al suggested that it is necessary to communicate the value of a scale-up to both leadership and implementers, ideally by providing real-time data from one scale-up phase to garner support for the next phase [16]. Among implementers, hospital administrative staff, in particular, reported a lack of engagement or opportunity to contribute to developing the scalable unit, which negatively affected their *“sense of ownership of it”* (Participant 37). In addition, CRO staff commented that:

...there are meetings and different groups but...the administrative end is not heard all the time...they come looking for secretarial support but then no budget or nothing available.

Participant 23

Perhaps, stemming from this lack of consultation, there was an unmet expectation from CRO staff that electronic referrals were going to save them time.

Everything that’s been done electronically, it’s supposed to save time and resources but actually it doesn’t. It does exactly the opposite mostly.

Participant 22

While electronic referrals will reduce the administrative burden for CRO staff once the interoperability issues between *Healthlink* and PAS are resolved, in the short term, it increases the workload because they have to manage an additional mode of referral request, in addition to traditional post and fax.

Furthermore, participants reported that the strategy for communicating the value of electronic referrals to GPs requires clarification to increase the proportion of GPs submitting electronic referral requests. This element of the scalable unit was not specified for the NERP step 1, and therefore, nobody was officially tasked with the responsibility to increase the proportion of GPs using electronic referrals. A hospital-based implementer suggested that:

I felt like why was I having to try to promote Healthlink?...Nobody could give me any communication tools to use for the GPs—so we had to try and figure out the best way to do it.

Participant 29

National-level participants suggested that ultimately, GPs are *“independent sole traders”* (Participant 27). The key authority capable of shaping the referral behaviors of GPs are the hospitals receiving those referrals.

That is really, a HSE hospital led kind of initiative...[The] ultimate step would be for management to say, “this is how we want you to refer”...unless there is an exception.

Participant 27

In this study, participant GPs broadly agreed with this perspective, suggesting that local hospitals are in the best position to change GPs’ referral behaviors together with local peer promotion through the ICGP’s Continuing Medical Education meetings. GPs commented that:

I think it would be great to see the hospitals running with the ball on this one alright.

Participant 13

They also said, *“...people listen to their peers”* (Participant 39). These comments illustrated the importance of acknowledging GPs’ professional independence in the Irish health system, when designing a communication strategy to engage GPs in the scale-up of a national IT program.

### Test Scale-Up

Test Scale-Up is where the underlying theory of change and the change package are tested in a broader range of settings to refine program hypotheses and build the belief and will of leaders and frontline staff to support the changes [16]. To progress the implementation beyond the NERP step 1, developing appropriate data collection and reporting systems (*Support System*) would require a more complete development of the scalable unit. Participants reported that electronic referrals present an opportunity to standardize what data are collected (Participant 7), especially through automatically populating demographic information (Participant 40], clinical history (Participant 22), and medication and allergy information [(Participant 29) from the GPs’ medical records. In addition, participants reported that electronic referrals are:

...giving greater visibility on referral volume, referral tracking, all those sorts of things by specialty within hospitals.

Participant 27

...whereas before we were relying on staff members putting them in an Excel...So now every referral to be processed must be on PAS.

Participant 5

The disadvantage of structured messaging is that it might limit GPs’ ability to communicate details about a referral. One GP commented that:

You can write a very good clinical note using free text, probably the best quality clinical notes because it captures what the patient and yourself are saying. You can’t do that with something that’s completely structured. When you’re picking from drop-down menus or whatever.

Participant 30

Moreover, participants highlighted the importance of buy-in from stakeholders on the type of data collected. One participant commented that:

I maintain that no clinician wants to work to a political target...They don’t mean anything clinically

Participant 35

A pilot participant cautioned that:

...when you use data in a punitive way...people are resistant to it.

Participant 19

These quotes highlighted the potential for electronic referrals to greatly improve the volume and quality of data collected on referral management as well as the importance of engaging with stakeholders to determine what data would offer the most constructive and meaningful insights for the quality improvement.

Regarding reporting systems, participants reported receiving a monthly *Healthlink* escalation report, showing electronic referral requests that were received by the hospital but for which no triage outcome had been logged on *Healthlink*. Although logging the triage outcome to *Healthlink* was beyond the scope of the NERP step 1, participants reported that this report supports CRO staff in tracking and tracing electronic referrals.

It has actually highlighted that we weren’t doing it [managing referrals] as well as we thought we were doing it.

Participant 22

Participants described two other national programs to which they submit data and receive reports relevant to electronic referrals, namely the HSE’s Outpatient Services Performance Improvement Programme (OSPIP) and an independent statutory body called the National Treatment Purchase Fund (NTPF). Crucially, however, neither OSPIP nor NTPF targets are formally aligned with any specific targets for the NERP step 1. A CRO participant commented that “in the Health Service, there’s no picture of what’s happening” (Participant 22).

This suggests a lack of data feedback to implementers, either on the NERP step 1 on its own, or a more strategic data reporting system that utilizes the data collected across HSE and statutory programs.

This lack of development in data collection and reporting systems exerts knock-on effects on the “learning system” (*support system*) for the NERP step 1. “Large-scale change requires a mechanism for collecting, vetting, and rapidly sharing change ideas or interventions”...to assemble a “change package” for scale-up [16]. Participants interviewed indicated that informal learning from the pilot sites was encouraged by the national implementation team, but no evidence emerged of any formal learning system. A national-level stakeholder commented that as part of the “go-live” training, it is:

...normally suggest[ed] that they [implementers] speak to other counterparts in other hospitals that have already gone live.

Participant 18

Furthermore, one implementation site participant commented that he had *“two conference calls”* (Participant 21) with a member of the pilot implementation team to learn from their experience, but otherwise, the data did not suggest that a learning system was in place for the NERP step 1.

Besides these support systems, an important *adoption mechanism* at this phase is a “culture of urgency and persistence” (*adoption mechanism*), motivating stakeholders to take action and sustain their efforts to take the program to full scale. A troubling theme emerged around how legacy IT failures have created a culture of caution rather than urgency for national health IT programs. One pilot participant explained that *“there’s the legacy belief around HSE ICT projects fail”* (Participant 38), and a national-level participant referred to how:

...some of them are not open to new stuff because they’ve been burnt in the past...Most sites need reassurance as to the impact it’s [NERP step 1] likely to have operationally for them.

Participant 18

Conversely, CRO participants reported that while they were cautious about electronic referrals, now that they are using *Healthlink*, they find it very straightforward to use, and there is a strong appetite for the implementation of an end-to-end electronic referral solution. In addition, one participant commented that:

Now that we know how easy it is to go electronic, it would be amazing to cut out all the filling.

Participant 4

Similarly, another CRO participant commented that:

...rather than sitting on this for a year and everyone would just get too complacent with it and then it’s more change...If you’re in the middle of a project and there’s more coming on board, you just take it.

Participant 25

These comments highlighted the importance of developing a complete, scalable unit, whereby participants are clear on what the vision for full scale is and they can then maintain momentum in going to full scale.

### Go to Full Scale

Go to Full Scale is the fourth and final phase of the *Framework for Going to Full Scale*. This is the rapid deployment phase in which a well-tested set of interventions, supported by a reliable data feedback system, is adopted by frontline staff on a larger scale [16]. “Design for sustainability” is a critical *adoption mechanism* for reaching this fourth *phase of scale-up,* whereby throughout the 3 activity phases (Develop the Scalable Unit, Test Scale-Up, and Go to Full Scale), the learnings about sustainability are used to refine the change package.

Policy uncertainty is a key sustainability issue for the NERP step 1, which has already been described above as the uncertainty about whether to proceed with the sociotechnical process changes for electronic referrals at the hospital level [25] or to postpone process changes until the hospital group CROs can be established [24]. An associated sustainability issue is the reconfiguration of administrative staff to work within newly established CROs. Historically, each hospital consultant would have their own secretary who manages referrals sent to that consultant. A national-level participant suggested that the consultant-level referral management creates *“a lot of duplication”* because secretaries *“wouldn’t be at full capacity all the time”* (Participant 28). Such a reconfiguration of staff is perceived as a challenge at the implementation level, where participants explained that:

...resources are still an issue with the Central Booking and the Central Office. So, to do it from within your current compliment [of staff] initially is difficult.

Participant 5

Similarly, another CRO participant commented that:

We do [have a CRO] only we have no one to sit in it. That’s why it comes to me. I’m the central office.

Participant 40

The variation in terminology used by participants to refer to the CRO in the above quotes reflects the variation in set-up and functions of these offices across sites. This variation helps to explain why some hospitals experience greater difficulty than others in implementing electronic referrals, if their administrative staff has not been reconfigured into a CRO.

The third key sustainability issue for the NERP is the persisting interoperability issues between *Healthlink* and the hospital PAS. One national-level participant explained that hospitals that have been upgraded from the old PAS to an Integrated Patient Management System (iPMS) can be integrated with *Healthlink*. The HSE Integrated Patient Management System (IPMS) team is working to *“incorporate that functionality...but again, it's just purely staff dependent”* (Participant 32) as this team does not have the human capacity to keep all hospitals technologically and procedurally up-to-date with the latest version. A CRO participant claimed that:

...it is a great system (new iPMS)...if the correct processes were in place, it would be perfect.

Participant 2

The process changes involved in implementing iPMS require CRO staff training. CRO staff reported that while the HSE IPMS team did train on-site trainers, these *“trainers only had a short period of time to get trained themselves”* (Participant 22), and as a consequence, the training *“wasn’t specific to your job, it was a general training group everyone went to”* (Participant 2). Moreover, upgrading hospital PAS to iPMS and providing the necessary training to CRO staff on how to use this upgrade are objectives beyond the scope of the NERP step 1. It is important to highlight, however, that to progress beyond the step 1 of the NERP (ie, eTriage, eConsult, eAppointments, ePrescribing, and eDischarge), this interoperability issue must be addressed.

Furthermore, consultant engagement was beyond the scope of the NERP step 1 because electronic referrals were printed once they reached the hospital. Implementing a more complete electronic referral solution will require hospital consultants triaging electronic referral requests online. An implementation site participant explained that they have had consultants from certain specialties requesting that *“their own referral form”* be accommodated within *Healthlink* to enable the collection of specialty-specific information to inform triage decisions. The *“way we got around”* that was by saying:

...well Healthlink said they would take on certain forms but if we could just run with this...and see how we get on with it.

Participant 21

Similarly, a national-level participant commented that:

...we get a consistent message from the hospitals that they’d like to do more in the way of specialist referral.

Participant 18

The challenge is that consultants throughout the country *“have to agree with those extra parameters* [for the electronic referral template] *that are unique for that speciality”* and then *“avoid a scenario that says well actually ideally we’d like 20 extra parameters from a GP”* (Participant 18). An implementation-level participant commented that:

I know that Healthlink did have some issues with [specialist referral forms]. They just want to consolidate as much as possible.

Participant 21

The *Healthlink*’s reluctance to integrate multiple specialist forms may, however, be driven by technical and financial obstacles to working with GP software vendors rather than a concern about whether or not GPs would have the time to complete specialist referral templates. Some national-level participants suggested that:

Engaging with the vendors is a challenge...because we’re very reliant on them doing the initial work to get their products modified.

Participant 12

*Healthlink* worked with the ICGP’s national General Practice Information Technology (GPIT) group *“to do a specification for the vendors...and coming up with agreements on cost and implementation time-frames”* (Participant 12). For *Healthlink* to integrate specialist referral templates, it would again need the support and cooperation of the ICGP GPIT group to engage with GP vendors to make the necessary upgrades. If specialist referral templates are perceived by GPs as unnecessarily burdensome, the ICGP GPIT group might not be willing or able to support this integration work in future.

## Discussion

### Principal Findings

This study aimed to theoretically frame the lessons learned from the NERP step 1 on the design and implementation of a national health IT program. The NERP step 1 presented an interesting case study of implementing a national health IT program because it explicitly committed to a technical-first implementation rather than a sociotechnical approach. A key strength of the program was that it was welcomed by most key stakeholders as the first step in the implementation of electronic referrals, delivering important patient safety benefits. A national implementation of electronic referrals was progressed, despite limited resources and outstanding interoperability issues. In addition, it gained credibility for a new *eHealth Ireland* unit, which demonstrated that it can deliver national health IT programs. Conversely, the limitations of the NERP step 1 were that it was poorly integrated in the wider policy and quality improvement agenda of HSE. Moreover, the lack of specification of the sociotechnical elements of the scalable unit created challenges in communicating the scope of the program to key stakeholders and restricted the ability of program managers and implementers to test and refine the change package. Regarding design, the theory-driven analysis of the NERP step 1 highlighted that it is necessary to specify the sociotechnical elements of a national health IT program at the national level. Of note, these do not need to be specified in tandem with technical elements but do need to be specified quite early in the implementation process, so that the change package can be tested and refined as a set of interventions for implementing the scalable unit (technical and sociotechnical elements). These principal results are detailed and compared with prior work in the next section.

### Comparison with Prior Work

#### Strengths and Limitations of the Scale-Up of the NERP Step 1

The first research question posed by this paper asked what are the strengths and limitations of the scale-up of the NERP step 1 as a technical-only intervention. A key strength of the NERP step 1 is that it scaled-up the technical capability of GPs to submit electronic referral requests to at least one OPD specialty in all public hospitals. The four patient safety improvements reported by the NERP step 1 participants, including speed of transfer, more complete demographic information, legibility, and traceability, have been recognized internationally as key benefits of implementing an electronic referral solution [3,26]. Second, the NERP step 1 has maintained progress in implementing a national electronic referral solution between GPs and hospital OPDs beyond the pilot stage. This commitment to enact learnings from a pilot stage must be commended since eHealth initiatives have been described as “plagued by ‘pilotitis’, with many small initiatives sprouting without any real coordination or ability to scale” [27]. Specifically, in reference to electronic referral systems, Bouamrane and Mair have reported that deployment is often “slow and characterized by limited and localized uptake, or regional rather than nation-wide implementations” [1]. Driven by executive leadership [5] within *eHealth Ireland*, as well as GPs’ appetite and technological capability for electronic referrals, the NERP step 1 has gained a considerable foothold at a national scale. Latest figures from February 2018, for instance, indicate that 16,752 electronic referral requests were submitted by GPs to hospital OPDs (Multimedia Appendix 2), representing 22.5% of the overall number of electronic referral requests submitted to Irish hospital OPDs in that month (Multimedia Appendix 3) [28]. The third key strength of the NERP step 1 is that it was perceived by national-level participants as building “credibility” within the health system for a newly established *eHealth Ireland* unit. This finding is important because it supports Eason’s suggestion that when scaling national health IT programs, there are “many agencies involved in shaping the system that reaches the users. Each agency can be considered a locus for part of the decision making” [13]. That is, agencies like *eHealth Ireland* set the strategic priorities in the program design, but crucially, they then need to have the credibility to successfully engage implementation sites (GPs and hospital OPDs) to adopt the technology. Huang et al flagged that the implementation of national health IT programs can just as easily fail at this institutional level as it can if the technology is not accepted by the end users [29]. This credibility is particularly important in the context of participant references to a “legacy belief that HSE ICT projects fail.”

Associated with this institutional complexity, a key limitation of the NERP step 1 was that it was poorly integrated within the wider policy and quality improvement agenda of the health service. The program was designed and implemented by *eHealth Ireland* to achieve a technical objective. National-level participants described the program as a separate piece of work to the HSE’s OSPIP, which is responsible for service improvement more generally within hospital OPDs. Greenhalgh et al cautioned that postponing the collaborative, cross-institutional work needed to deliver a sociotechnical implementation only increases the chances that a technical system will be met with resistance from other stakeholders [26], particularly if the aims of the program do not align with the professional norms of the end users [30]. In addition, Huang et al suggested that major health sector innovations typically “emerge from negotiations between diverse stakeholders who compete to impose or at least prioritize their preferred version of that innovation” [29]. Instead of a top-down approach to technology deployment, Coiera advocated for a “middle-out” approach to developing national health IT systems, whereby technical goals are set to help achieve clinical or service standards [31]. These standards are not static, and therefore, a partnership approach is required between health care providers (clinicians and managers) and government and the IT industry to constantly develop national health IT systems in line with health service priorities and the evolving potential of technology. Under this approach, “implementation never stops” [31] and implementing technical capability as an objective separate from a specific clinical or service target would not be pursued.

The second, related limitation of the NERP step 1 was the incomplete specification of the program’s scalable unit. Once all hospitals had at least one OPD specialty accepting electronic referral requests from GPs, the single objective for step 1 of the program was achieved. In this study, participants reported an implementer burden owing to this lack of specification. Hospital participants reported having to try and figure out for themselves how to engage local GPs, although they did not consider this their responsibility. Some hospital administrators expressed dissatisfaction with the low level of consultation, which inhibited them from communicating the “double-jobbing” challenges associated with sending the GP triage outcome message. Sending this message was a feature of the pilot project but not the NERP step 1; however, interviews with hospital administrators suggested that the scope of the NERP step 1 was neither clearly specified nor communicated to them. This limited program specification also restricted the potential to develop data collection and reporting systems, through which individual implementation sites could monitor their progress [32]. If the program had been better integrated within the wider quality improvement agenda in the HSE, a broader range of mandatory clinical or service targets could have been set, as was the case in the rollout of electronic referrals in Scotland [1]. Importantly, as described by participants, clinicians do not wish to work to “political targets.” Any additional targets set must be patient-centered to ensure that the learnings gained from the data are meaningful for various stakeholders [26,33].

#### Going to Full Scale With a National Health IT Program

The second research question asked whether the sociotechnical elements of a large-scale national health IT program need to be specified at the national policy level. Analyzing the NERP step 1 using the *Framework for Going to Full Scale* [16] revealed that the scalable unit for the NERP step 1 did not include a minimum specification of the sociotechnical elements, and critically, this incomplete specification of the scalable unit inhibits the scale-up of the NERP step 1. The one element specified for the NERP step 1 was that all publicly funded Irish hospitals would be set-up with the technical capability to accept electronic referral requests. No specification was provided for the proportion of OPD specialties targeted, the proportion of electronic versus paper referral requests targeted, or the proportion of GPs targeted to use electronic referral requests. Consequently, data collection and reporting systems were not put in place to capture what change package was used to implement these elements of the scalable unit at each implementation site. For example, OPD specialties requiring a specialist referral template instead of the standard GP-OPD referral template remain unclear. For NERP to progress to the Test Scale-Up phase, a more complete scalable unit needs to be formally specified. In addition, data collection and reporting systems need to be put in place to monitor progress in implementing this scalable unit, and a learning system established to utilize the data collected. The learning system synthesizes the lessons from early implementation sites to guide future implementation sites on the design of a well-tested change package. The answer to this second question, therefore, is yes, the sociotechnical elements of a large-scale national health IT program do need to be specified at the national policy level if it is to progress to full scale. Importantly, it is the “scalable unit” (ie, goals of the program) and not the “change package” (ie, the set of interventions) that needs to be specified at the national level, and therefore, it is not a one-size-fits-all approach to implementation which is advocated by this study or the *Framework for Going to Full Scale*. Furthermore, each implementation site is given the flexibility to undertake a local sociotechnical design of their “change package,” in that they can select a set of interventions that fit the priorities and circumstances of their local context. Ultimately, however, their implementation will be assessed nationally on the extent to which the scalable unit is delivered.

The wider literature is highly critical of designing national health IT programs with rigid top-down change packages that do not leave space for local adaption. Coiera, for instance, argued that centrally defined, top-down implementations of national health IT programs become increasingly out of step with service needs, and clinical providers will have to build workarounds to make the aging system meet emerging needs [31]. If emerging needs are left unaddressed, the workarounds will add unmanageable local variations to what was intended to be a singular national design [31]. Regarding a technical-only scale-up, a dynamic cost-benefit analysis study reported that the potential gains of implementing an electronic message exchange could be reduced by 40%-50%, if old working procedures to fit old technology are maintained after new technology is implemented [34]. This type of economic argument warrants careful consideration for the NERP step 1 in light of the outstanding interoperability issues between *Healthlink* and hospital PAS, which until resolved will require the “double-jobbing” of old and new working procedures. These arguments illustrate that the overall success of the technical elements of a national health IT program depend on their interaction and fit with the sociotechnical system.

As such, the academic literature’s advocacy of a sociotechnical approach to implementing national health IT programs is not contradicted by this study. This paper started with an observation that the NERP step 1 was initiated with a technical-only intervention and uncertainty about whether this type of implementation strategy [13] put the NERP step 1 at odds with the best practice, sociotechnical approach. The key learning from this study is that the implementation of a national health IT program requires an interaction and ultimately a fit between the new technical solution and the existing sociotechnical system. A program may be initiated with a technical-only intervention, similar to the NERP step 1; however, the priority for such an intervention is then to fit the technical elements of the program to the sociotechnical system within which it is being implemented. It is recommended that policy makers and implementers use a quality improvement framework such as Barker et al’s *Framework for Going to Full Scale* [16] to help guide them in the design and implementation of national health IT programs.

### Critique of the Framework for Going to Full Scale

Barker et al’s *Framework for Going to Full Scale* [16] informed a theoretically driven thematic analysis of the semistructured interview data. The concept of the “scalable unit” proved crucial to identifying the *phases of scale-up* achieved by the NERP step 1 and informing an understanding of the limitations of a technical-only scale-up. One challenge encountered in using this framework was that the distinction and interaction between the “scalable unit” and the “change package” are not made explicit in the original paper. Both elements are described as being generated at phase 2, Develop the Scalable Unit, with the scalable unit defined as “the smallest representative facsimile of the system targeted for full-scale implementation”; the change package is described as “a set of context-sensitive strategies and interventions” [16]. Having applied the framework to the NERP step 1, it is suggested that future applications of the framework might find it helpful to think of the “scalable unit” as the goals of a program, whereas the “change package” is the set of actions undertaken to deliver each goal. As such, both constructs are interdependent. As the scalable unit is developed, the change package for implementing that scalable unit needs to be tested and refined, and if the scalable unit changes, so must the change package. A comprehensive critique cannot be provided on phases 3 and 4, Test Scale-Up and Go to Full Scale, because the NERP step 1 did not reach these phases. In particular, it will be important for future applications of the framework to detail the process through which an implementation site selects, tests, and refines their change package (set of interventions), and at the national level, to explore the degree of variation in the change packages employed across implementation sites and the impact this variation has on delivering the scalable unit.

### Study Limitations

A key limitation of this study is that hospital specialists were not interviewed. Hospital specialists were not formally engaged in the design or implementation of the NERP step 1 and therefore were not considered key informants in this early stage of the program. Upon the receipt of an electronic referral request, the Hospital CRO prints the electronic referral request. By the time it reaches a specialist, it is a paper-based referral request, just like any other. The only change encountered by specialists is that electronic referral requests from GPs are presented to them for triage on a standardized template. The triage phase of referral management was not included in the NERP step 1, and therefore, specialist dissatisfaction or satisfaction with the standardized GP-OPD referral template was beyond the remit of this study. Issues were raised within the study, however, for which it would have been valuable to have obtained a specialist medical perspective. These include the centralization of the referral management to a CRO at a hospital or hospital group level or the suitability of using the standardized GP-OPD referral template for all OPD specialties. Hence, future research should focus on these issues as they relate to the later stages of the *phases of scale-up*.

The second limitation is that the participants were not recruited from randomly selected implementation sites. Access to 1 pilot site and 5 NERP step 1 implementation sites was arranged through *eHealth Ireland*. Although not randomly selected, the 5 NERP step 1 sites did include public and voluntary hospitals, as well as regional and urban hospitals, and so, a broad representation of implementation sites was achieved.

### Conclusions

This qualitative study of the early-stage implementation of the NERP provides empirical insights into the complexity of implementing a national health IT program. The incremental design of this program—with step 1 only seeking to scale-up the technical capability for the e-request phase of an electronic referral solution—made the NERP step 1 an interesting case study from a sociotechnical perspective.

The strengths of this implementation were that it successfully scaled-up the technical capability for GPs to submit electronic referral requests to at least one specialty in all hospitals in the Irish public health system. In addition, it maintained progress in the implementation of an electronic referral solution beyond piloting despite limited resources and outstanding interoperability issues. Finally, it built credibility and confidence in the new *eHealth Ireland* unit’s ability to successfully implement a national health IT program. Conversely, the limitations of this program were that it was poorly integrated within the wider quality improvement agenda of the HSE. The incomplete specification of the vision for full scale created uncertainty for stakeholders on their roles and responsibilities within the program, as well as a lack of clarity on the emerging change packages, which need to be tested and refined.

These limitations were a consequence of not specifying a complete scalable unit, including the sociotechnical elements of the program. In conclusion, although the sociotechnical elements of a program do not have to be specified in tandem with technical elements, they do need to be specified quite early in the implementation process so that the potential change packages for implementing the scalable unit can be tested and refined into a scalable set of interventions.

## Appendix

Multimedia Appendix 1 Number of electronic referral requests submitted to participating hospital outpatient departments in 2015/2016. Data collection period is Oct 2015-May 2016. Source: *eHealth Ireland*.

Multimedia Appendix 2 Monthly number of electronic referral requests (Sep 2015-Feb 2018). Source: *eHealth Ireland*.

Multimedia Appendix 3 Upward trend in % of general electronic referrals to outpatient departments (Mar 2016-Feb 2018). Source: *eHealth Ireland*.

## Abbreviations

CIO: chief information officer
CRO: central referral office
GP: general practitioner
GPIT: General Practice Information Technology Group (Ireland)
HIQA: Health Information and Quality Authority (Ireland)
HSE: Health Service Executive (Ireland)
ICGP: Irish College of General Practitioners (Ireland)
ICT: information communication technology
iPMS: Integrated Patient Management System
IPMS: Integrated Patient Management System Programme (Ireland)
IT: information technology
NTPF: National Treatment Purchase Fund (Ireland)
NERP: National Electronic Referral Programme (Ireland)
OPD: outpatient department
OSPIP: Outpatient Services Performance Improvement Programme (Ireland)
PAS: patient administrative system
PDSA: Plan-Do-Study-Act cycle of quality improvement
UCD: University College Dublin
