# Supplementary figures and images for: Implementing a National Electronic Referral Program: Qualitative Study
Source: JMIR Med Inform. 2018 Jul 18;6(3):e10488. doi: 10.2196/10488 (PMC6070727; doi:10.2196/10488)

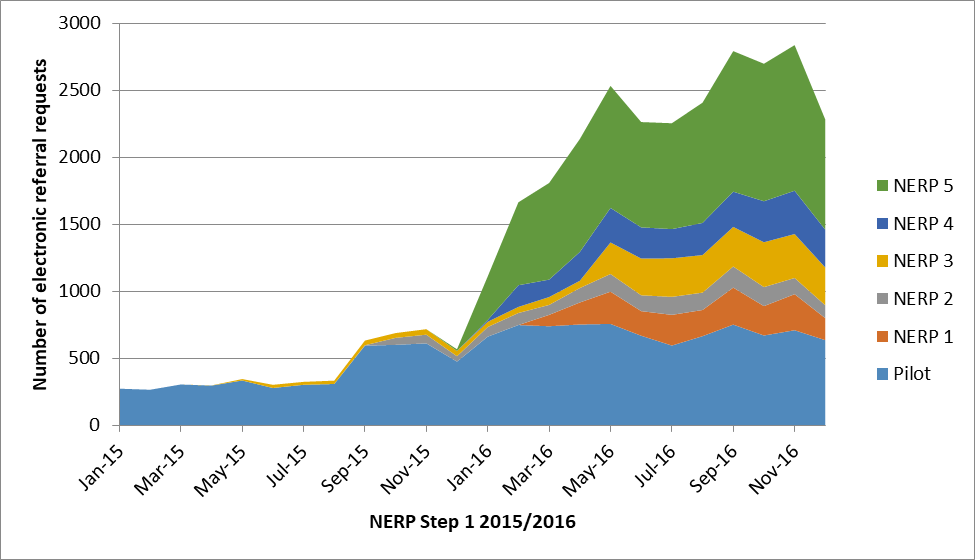

Supplement: Multimedia Appendix 1 [file medinform_v6i3e10488_app1.png]

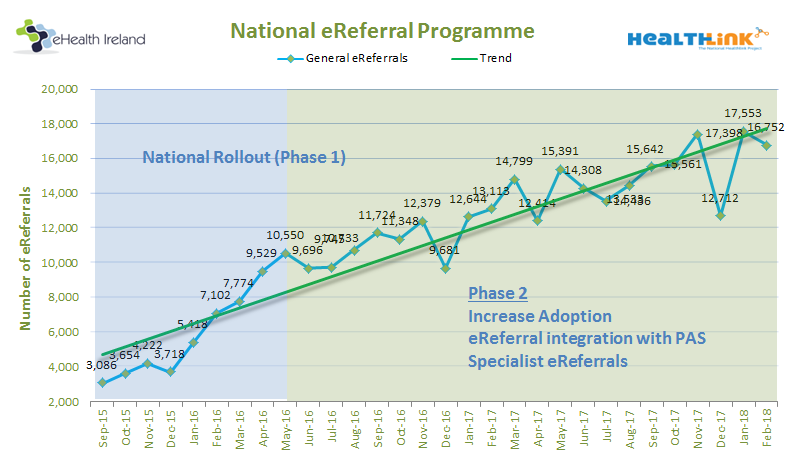

Supplement: Multimedia Appendix 2 [file medinform_v6i3e10488_app2.png]

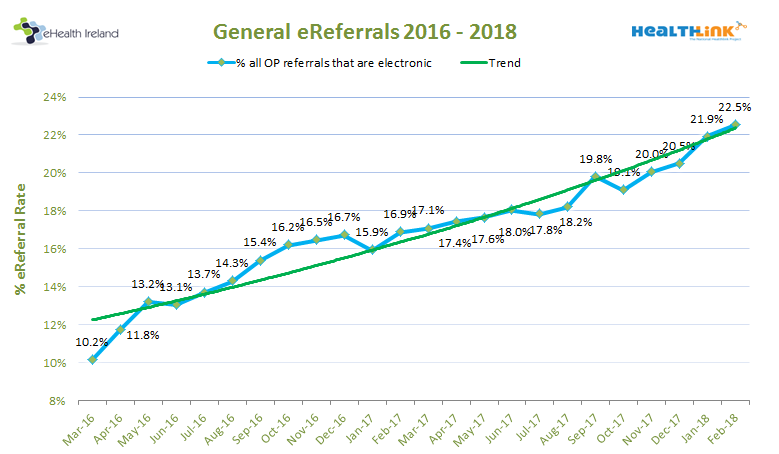

Supplement: Multimedia Appendix 3 [file medinform_v6i3e10488_app3.png]
